# Cause, Symptoms and Treatment of Cholera

**Published:** 1866-06

**Authors:** J. M. Johnson

**Affiliations:** Atlanta, Ga.


### ARTICLE II.

*Cause, Symptoms and Treatment of Cholera.*—By J. M. JOHNSON, M. D., of Atlanta, Ga.

The appearance of Cholera upon this continent, within the past few days, excites, as it should do, the liveliest interests, not only amongst medical men, but all classes of society, from the centre to the circumference of our country. Nor is this to be wondered at, since the oldest and wisest of our "noble profession" cannot contemplate the return of the pestilence to our shores with any other feeling than that of horror and alarm.

The irresistible speed with which it strides from continent to continent, the subtlety with which it evades quarantines and sanitary regulations traveling, with the speed of the railroad and steamship, it seeks first the crowded mart where, like the vampire, it sucks the blood unseen and unfelt; then starting again with the moving masses that throng our thoroughfares, large and small, it ramifies the whole country; and suddenly, at midnight, without warning, it smites its sleeping victims in distant towns and neighborhoods.

So mysterious are its laws, that the most earnest inquirer into etiological and pathological science has been unable to fathom them. So deadly its poison that the most powerful remedies, unless early applied, and with consummate skill, are as nothing. So sudden its attacks that the blood is dried up and the heart is sealed with the seal of death ere the approach of danger is realized. Learned observers have declared it contagious; equally learned observers have proclaimed it non-contagious. The public judgment staggers beneath the load of conflicting theories; and before any of them assumes the dignity of settled belief, the foundations are swept away, the superstructures fall, and disappointment and uncertainty are all that remains. What is to be done in such an emergency as this? Shall we give up for lost the whole question of the pathology and treatment of cholera? Must we acknowledge ourselves vanquished, and proclaim with the red man, "It is not cholera, it is death."

Its history, until within the present century, is unrecorded.

Since our day it has visited every part of the globe, except perhaps the frozen zone. It raged in Russia in the winter of 1830-31, during the coldest weather known for near a quarter of century, and tens of thousands fell victims to it. It prevailed also in the tropics, and in the temperate latitudes with equal severity two years later. It is not bounded by seas—adheres to no geographical lines—from a common centre it moves east, west, north or south, or all at once, but invariably follows the great commercial lines of the world, turning to the right or left to follow a railroad or navigable river, or even a stage line; it penetrates remote villages and settlements, bringing consternation, sorrow and death amongst the primitive and humble.

Its attacks are those of a consummate general. Heat and cold, summer and winter, wet or dry weather, all the agents of disease are its allies. The secrets of the constitution are found out, the salients, if not rendered impregnable by courage, temperance and prudence, are successfully assailed, and the citadel falls. The poor are its surest victims, being badly housed, and badly fed and clothed, and without the means or judgment to provide for their safety. Scarcely one found in the track of the pestilence escapes it, and more than three-fourths perish. If I were asked for the best sanitary means for this city, in view of the near approach of cholera, I would answer unhesitatingly look first to the condition of the poor—see that their tenements, in addition to being clean, are properly ventilated and lighted—fresh air and sun light are indispensable—furnish them wholesome food, by a public tax if necessary, and compel them, by proper penalties, to observe strictly the hygienic and sanitary rules laid down for their government. I would shut up all the beer and liquor shops, forbid exposure to night air, or the use of green corn, cabbages, cucumbers, melons, radishes, etc., etc.; compel the use of good clean water, and I would prohibit town meetings, picnics, etc., from the well known fact that masses are sure, where the cholera atmosphere exists, to breed the disease, whilst individuals who keep away can scarcely be considered liable to it.

I would see that the streets, and wells, and sewers, and privies, and stables were thoroughly cleansed out, and that the chloride of lime was freely used every day, on every lot in the city, that was inhabited.

Cholera is a disease which destroys the homogeneity of the blood. Whatever may be the cause, the effect at least is known. It is not inflammation, nor yet is it a fever—it is *cholera.* The vomiting, and purging, and cramps, are symptoms only. The circulating mass is the seat of the trouble, no matter what its cause. The patient may have had no premonition of the approach of the disease—he has perhaps labored, and eaten, and slept as usual, but at mid-night he is aroused from his slumbers with an irrepressible vomiting and purging. The first effort empties both the stomach and bowels of their contents. In a few minutes both vomiting and purging is renewed, and rice water discharges begin. This is serum, the watery part of the blood; as fast as it is thrown out, the absorbants take up from the surrounding tissues an additional supply, which on the instant is thrown off as effete matter.

This process of accumulation and waste goes on from two or three to a dozen hours—just in proportion to the violence of the symptoms, and culminates in the total exhaustion of the last drop in the system. The circulating vessels contain nothing but glairy tenacious clots glued to their walls, or rather gluing their walls together, the full round muscles of a man, in the vigor of manhood are reduced to a mere tendinous sheath adhering closely to the bones. The skin and areolar substance in like manner drained of the last drop of fluid, adheres to the shrunken attenuated muscles as closely as if drawn there by main strength. The brain, all the cavities of the skull, the eye balls, the spinal cord, glands, in short, every fibre and tissue is wasted, and contracted to an extent utterly incredible, and beyond the power of belief, and this not unfrequently occurs in less time than it takes to eat a fashionable dinner!

I first saw and treated the cholera in New Orleans, in 1833. In June of that year I left the city to return to Kentucky. Two gentlemen from Louisville occupied the state room immediately in front of me. We retired to bed at mid-night, all in perfect health so far as known. I arose at six o'clock next morning, and Mr. Semple, one of the gentlemen named, had been dead some time, was shrouded and laid out, and his friend was dying—ten deaths occured that night, the first after leaving port, and none lingered longer than six or seven hours.

I have not attempted to give the etiology of cholera, nor will I. There are secrets in Heaven, and all nature abounds in them. Man has been endowed with wonderful capacities, and the great law of his being is to move forward. Not content with what is known, he must pass on to new fields of discovery, calling into requisition every faculty Divine Providence has vouched safe him. Yet unappalled by the difficulties which the subject presents, and I trust without ostentation, I approach the great pathological questions. "What is cholera?" What part of the complete organism is involved? and what the true indications of cure?

Cholera is a scourge. Like other epidemics, it is a minister of the destroying angel, turned loose to "hurt the world for a season." In vain have theorists speculated upon its origin and cause; one after another these well built up fabrics have had to yield to the logic of events, and gave place to new data and new facts. And yet the true pathologist moves on with tireless zeal and endless hope. There is a living irrepressible sentiment, the very heroism of courage, that takes him to the cell of the dying convict, and the infectious hovels of the poor, defying obstacles and danger, and even death; his errand is one of discovery. Philosophy whose shield is truth, accompanies him; Science lends the light of centuries; Etiology and Pathology cast their treasures at his feet; Fame stands ready with scroll in hand to record the grand discovery, the cause of cholera. After rising step by step, higher and higher, from plane to plane, every law and every principle that the human mind can comprehend, has been brought into requisition, the problem remains unsolved; one word records the result—undiscovered.

I have intimated elsewhere that cholera was a blood disintegrating disease. Certainly it cannot be denied that the only traceable primary lesion is in the circulating mass. It will not do to attempt to account for cholera upon the hypothesis of irritation in the intestinal canal; this being nothing more than a symptom. We must look beyond this for the intrinsic cause.

Having said that cholera was a lesion of the blood, I will give my reasons very briefly for this opinion. The universality of certain agents will be admitted by all. The atmosphere is the same everywhere, and liable to the same temporary changes every where. The earth being the home of electricity, it is alike all pervading. And whilst we know but little comparatively, of this extraordinary agent, enough is known to prove its power in the production and perfection of life. Whilst we would hardly be warranted in saying it is the vital principle, few who have investigated the subject would dare to say it is not. It courses through the brain and nervous trunks, unseen and unfelt, imparting health and vigor to the constitution, and courage and will, and manhood to the character, in proportion as the amount is large and harmonious, or cowardice and servility, where it is small. The former attain the greatest longevity, originate and carry forward all great enterprises, head armies, establish kingdoms, and set up kings. They abound in every department of life and business, with diversity of tastes and pursuits, but the same success attends them. From the centre of the scale, between the highest and lowest, upward and downward, there is a marked difference in character. From the middle upward you have the men who are the soul of the social state, who control society, and subordinate its members to their proper avocations and pursuits. Below this you have a restless, disturbed element, incapable of organizing themselves into communities, that mutual benefit, progress and happiness may result. Wisely, therefore, society is made up of all classes; the weak to be protected by the strong, the bad to be kept in check by laws founded on great moral truths, the guilty punished, that virtue may be vindicated, etc. The first class are not liable to contract disease, except as the consequence of willfully wasting the powers of the constitution—their intuitions leading them to just conclusions as to the place and mode of safety. But it is very different with the other class; they readily take on all the forms of disease, and it is with them that the harvest of death begins and ends. With this class fear predominates, and unreasoning alarm and *panic* follow.

If this view is tenable, will it be illogical to conclude that one chief extrinsic cause of cholera is panic? Cholera is acute diarrhœa with congestion. But waiving for the present both its extrinsic and intrinsic causes, let us turn our attention to the people and country from whence it comes, in its malignant and epidemic form. Its history is that it originates amongst the pilgrims of the east, while visiting in great numbers their sacred places for the performance of certain religious rites, and in fulfilment of certain vows. It is asserted that they are ignorant, superstitious, filthy and destitute in the last degree. Thousands perish from hunger, fatigue and exposure, as well as from disease. What disease so likely to prevail, under the circumstances, as acute diarrhœa? My experience while connected with the army, proves that the collection of men into large masses, especially those from rural life, even under the favorable aspect of comfortable quarters and good food, brought on nearly all forms of disease, but especially acute diarrhœa. Reasoning *a priori*, the degraded masses of the eastern world, where the epidemic originates, would be equally liable to it, to say the least, but without our hygienic and sanitary means, or skillful medical advisers to cure and arrest the progress of the epidemic. The history of cholera further proves that none regard it with more alarm than do the people of India. A panic prevails throughout the country as soon as the appearance of the pestilence is known. Need I introduce proof to show that enlightened Europe and America regard it in a no less serious light?

If, therefore, panic is an extrinsic cause of cholera, and the intrinsic cause functional disorder of the nerves and lesion of the blood, what is the essential bodily change pathognomic of cholera? I think myself justified in assuming that cholera begins in the cerebrum pneumogastric, and great sympathetic ganglia. The great tri-splanchnic filaments being nerves of sensation, and distributed as they are through the three great cavities, their functions, no less than their influence, is all pervading. Under the influence of cholera panic, their functions are either partially or wholly withdrawn, the will is disturbed, the appetite fails, the breathing is constricted, the heart's energy is impaired, the blood accumulates in the veins, the stomach and bowels become irritable, and a feeling of helpless exhaustion sets in, the nutritive functions are suspended, and chyle and lymph no longer supply nourishment to the blood. These are some of the phenomena attendant upon partial suspension of nervous function. If there is total suspension, attended with reflex action of these nerves back upon their centres, the symptoms will be greatly intensified and the result more rapidly, and more certainly fatal.

The reader will please bear in mind that I am only discussing the question with reference to its bearing upon the subject, of the intrinsic cause of cholera, and not the general result of the disturbance of the functions of these nerves as they appear in every day practice. I know we can trace syncope, chorea convulsions, hemaplegia, hysteria, etc., etc., to a like cause, but not generally from a like cause.

We turn now to the morbid changes wrought upon the circulating mass. There are living active poisons enough in the system to cause death at any time, but for the rapid and ever-flowing current, which ceaselessly performs its circuit, and throws off by the lungs, liver, etc., all such noxious accumulations. But suspend its functions, even for a few minutes, clots form in the heart, and great venous trunks, and death follows. If the breathing is only partially suspended, carbon accumulates in the blood and acts as an irritant poison. It is therefore capable of demonstration that there are poisons locked up in the system, capable of destroying it, if only one function is disturbed. If, in addition, you have partial cerebral syncope, generally present in instances of panic, or any other excessive emotional cause, by which the nutritive functions are impaired or cut off entirely, the breathing disturbed, and the emotional nerves having ceased their functions, there will be increased action of the pneumogastric and great sympathetic, to be followed most likely by reflex action back upon their centres, and thus disturbing the whole system of nerves, and by this means, every function of the body.

The particular cause that disturbs the homogeneity of the blood is difficult to trace. Autopsy discloses the fact that the arteries are empty ,and that inspissated fibrinous lymph fills up the minute veins, and thereby prevents the return of the blood to the arteries. All of the veins are found to contain this substance. The nervous trunks appear to have suddenly ceased their functions, but present no other changes except loss of fluid. The stomach, bowels and liver show scarcely any traces of inflammation. The cause of death is the loss of the watery part of the blood; the blood must therefore be the objective point.

*Treatment.*—The earlier symptoms, known by the designation of cholera, is simply a lax; at this stage, but little treatment is required. The patient should be placed in bed, his feet bathed in hot water. He should take a cup of hot tea, or hot brandy toddy, with 30 drops of laudanum, to be repeated every time the bowels are moved. Also sulphuric acid, one part, water, twenty parts; of this, one teaspoonful every hour. Mustard should be placed over the stomach and bowels, and also over the chest. A liniment composed of equal parts of aqua ammonia, camphor, turpentine and sweet oil, should be applied to the spine every hour or two, as hot as it can be borne. This should be applied with a flannel cloth, and with as much force as can be borne, without rubbing the skin off. When the purging ceases, if the skin is warm and the pulse full, the remedies may be discontinued, but the patient must remain in bed, from two to six days, and the diet should be the most simple and nourishing, the quantity for the first day or two almost infinitesimal: coffee, tea, fresh boiled milk, chicken tea, buff tea, etc., etc.

A vast majority of cases, taken in the incipient or premonitory stage, will be radically cured by this treatment alone. Quiet has much to do with the recovery of such cases, and cannot be too strongly enjoined. I have seen many cases after they were apparently cured, leave their beds, relapse and die in a few hours. A relapse is almost certain death. Opium and stimulants should be discontinued as soon as possible; the sulphuric acid may be continued longer. Brandy and opium will have to be administered throughout the active symptoms, but great care must be taken to avoid the bad effects of both. I have seen many patients die, after cholera had been arrested, from congestion of the brain, superinduced by these remedies. From a teaspoonful to a tablespoonful of brandy every fifteen or thirty minutes is enough, and from twenty to forty drops of laudanum, or one or two grains of opium every hour, will generally meet the indications.

In the second stage of cholera, where you have the rice water discharges, add to the brandy and water two and a half grains of carb. of ammonia, to each portion of brandy and laudanum, and continue applications to the spine, and mustard to the bowels and give the following pill:

℞ Calomel, grs. x.
Quinine, " xx.
Make pills, vi.

Give one every hour. If there are cramps, and the symptoms continue to intensify, discontinue the laudanum from the mixture of brandy and ammonia, and give the following:

℞ Chloroform, ʒ ii.

Laudanum, ʒ ii.

Sulph. Ether, ʒ ii.

Spts. Camphor, ʒ ii.

Mucilage Gum Arabic, ʒ ii.

Mix.

Give one teaspoonful every ten, twenty or thirty minutes, and if necessary, double, or triple the portion. Also, use the following injection:

℞ Nitrate Silver, grs. x.
Aqua Distillae, ℥ i.

Throw this up the bowels by means of a flexible tube, as far as it can be passed, and repeat it every time the bowels are evacuated.

The patient must be inspired with confidence. Brandy must be used to restore the cerebral functions; frictions over the spine, to act upon the great cerebro-spinal centre, for the restoration of functions of the pneumogastric and sympathetic nerves; mustard to the chest, to stimulate the minute nerves and capillaries, and excite muscular action to restore the breathing—to the stomach because it is the centre of vital action, and revulsives assist powerfully in the restoration of its functions, and over the heart, liver and bowels, for the same reason, and all at once because the larger the surface covered, the more certain and speedy the reaction.

In addition to giving carb. ammonia as a stimulant, it is very generally admitted to be the agent by which the fluidity of the blood is maintained. Is it not probable that its influence is exerted upon the fibrinous lymph, by which the union of the different elements are perfected and kept up?

Allow me to say, in conclusion, that I have seen a great deal of cholera, first in 1833, in New Orleans, then in Kentucky, in 1849, and again in 1853-4. The plan of treatment given above was not adopted by me until the last visitation of cholera, in 1853, and again in the spring and summer of 1854. It was suggested by a conversation with Dr. John Berry, of Uniontown, Ky., a man of talent and experience—nor did I lose a patient after I adopted it.

